# Disequilibrium, Adaptation, and the Norse Settlement of Greenland

**DOI:** 10.1007/s10745-018-0020-0

**Published:** 2018-09-10

**Authors:** Rowan Jackson, Jette Arneborg, Andrew Dugmore, Christian Madsen, Tom McGovern, Konrad Smiarowski, Richard Streeter

**Affiliations:** 1 Geography, School of Geosciences, University of Edinburgh, Drummond Street, Edinburgh, Scotland EH8 9XP UK; 2 Department of Archaeology, School of Culture and Society, University of Aarhus, Moesgård Allé 20, 8270 Aarhus, Denmark; 3 Middle Ages, Renaissance and Numismatics, National Museum of Denmark, DK-1220 Copenhagen, Denmark; 4 Human Ecodynamics Research Centre & Doctoral Program in Anthropology, The Graduate Center, City University of New York, 365 Fifth Avenue, New York, NY 10016-4309 USA; 5 Hunter Zooarchaeology Laboratory, Department of Anthropology, Hunter College, City University of New York, 695 Park Ave, New York, NY 10021 USA; 6 School of Geography and Sustainable Development, Irvine Building, University of St Andrews, St Andrews, KY16 9AL UK

**Keywords:** Greenland, Norse, Niche Construction, Culture, Climate, Disequilibrium

## Abstract

There is increasing evidence to suggest that arctic cultures and ecosystems have followed non-linear responses to climate change. Norse Scandinavian farmers introduced agriculture to sub-arctic Greenland in the late tenth century, creating synanthropic landscapes and utilising seasonally abundant marine and terrestrial resources. Using a niche-construction framework and data from recent survey work, studies of diet, and regional-scale climate proxies we examine the potential mismatch between this imported agricultural niche and the constraints of the environment from the tenth to the fifteenth centuries. We argue that landscape modification conformed the Norse to a Scandinavian style of agriculture throughout settlement, structuring and limiting the efficacy of seasonal hunting strategies. Recent climate data provide evidence of sustained cooling from the mid thirteenth century and climate variation from the early fifteenth century. Archaeological evidence suggests that the Norse made incremental adjustments to the changing sub-arctic environment, but were limited by cultural adaptations made in past environments.

## Introduction

There is increasing evidence to suggest that human and ecological communities do not follow uniform responses to climate-induced stress (Cumming *et al.* 2008). Studies of cultural and ecological change in the Arctic have uncovered extended periods of disequilibria, where biota and hunter-gatherer communities followed non-linear responses to climate volatility (Normand *et al.* 2013; Riede and Pedersen 2018). We here evaluate the role of social-ecological disequilibria in shaping the demise of the Medieval Norse settlements in Greenland. Using insights from Niche Construction Theory (NCT), we argue that environmental modification had a significant role in the Norse demise, not because they degraded their environments, as some have argued (i.e., Diamond 2005), but because they were culturally invested in transported “domesticated landscapes” (Terrel *et al.* 2003: 323) that limited their capacity to adapt to climate change.

Scandinavian Norse settlers introduced agriculture to Greenland in the late tenth century, importing an ‘agricultural niche package’ of non-native domesticated animals and plants tuned to Norwegian and Icelandic environmental conditions (Riede 2011). Early settlers cleared willow and birch scrub to extend grasslands for grazing livestock, creating synanthropic landscapes with impacts on vegetation, soil, and landscape structures (Dugmore *et al.* 2005). The introduction of agro-pastoralism accelerated soil erosion, but this is far more isolated than often reported and had a limited impact on home-field hay production (Golding *et al.* 2011).

We focus on the biocultural relationship between Norse farmers, their cultural landscapes, and the changing biophysical conditions in Medieval Greenland. Synergistic changes to climatic stability and European markets operating at regional scales had significant long-term impacts on local-scale resilience (Dugmore *et al.* 2012). Anthropologists and cultural geographers have explored the detailed relationships between culture and landscapes in different ways, from the cultural-semiotic relationships that form common identities to the interactions shaping social practices and values (Berkes 2017; Brace and Geoghegan 2011). Biological and physical impacts on landscapes often have significant cultural impacts—eroding social institutions, traditional practices, and iconic habitats (Adger *et al.* 2013). We argue that the translocation of the Norse ‘agricultural niche’ from stable environments in Norway to the different and less predictable ecologies of Greenland was responsible for social-ecological disequilibrium and the decline of Norse settlement.

### Historical Ecology, Macroevolution, and Niche Construction Theory

Historical ecology—the study of long-term interacting human and natural processes manifest in landscapes—is a cross-disciplinary field connecting ‘climatic and biotic variability (such as wildlife grazing, browsing, and fire) in the context of human land use and management’ (Armstrong *et al.* 2017: 8; Crumley 1994). NCT raises questions of how the dispersal of domestic species across the globe has affected landscape and seascape ecologies and how this influences human capacities to adapt to environmental change (cf. Armstrong *et al.* 2017).

The colonisation of new environments involves a process of ‘landscape learning’ wherein initial settlers acquire ecological information about resource location and timing, limitations of the environment (i.e., carrying capacity) and sustainable social organisation (Rockman 2003). Constructing sustainable social-ecological systems can be constrained by information about long-term eco-dynamic changes and the historically contingent ecological knowledge of new settlers (Dugmore *et al.* 2009). The human capacity for social learning through language and symbol systems ‘vastly increases the fidelity of information transmission, making it possible to modify and fine-tune these behaviours’ (Zeder 2016: 332). In macroevolutionary theory, the cultural transmission of information is a fast-operating process and enhances adaptability to new environmental conditions by constantly updating knowledge, practice, and belief systems (Berkes 2017). This information can be transferred between generations in art, objects, myth and legend, and ritual performances to give meaning to local environments known as traditional ecological knowledge (TEK) (Riede 2012).

Niche construction (NC) can be defined as behaviours whereby organisms, or agents, modify environments in such a way that ‘selective pressures’ acting on organisms are changed (Odling-Smee *et al.* 2013). NC is divided into ecological, genetic, and cultural inheritance domains to explain the ontogenetic processes of human environmental modification (‘ecological inheritance’) and gene-culture co-evolution by modified natural selection (‘genetic inheritance’). Knowledge of niche-modifying behaviour is stored in cultural-spiritual practices (‘cultural inheritance’) to enhance the survival of future generations (Smith 2011). Storage of historical information in cultural traditions is essential, especially in agricultural economies where information transfer is vital to making predictions about timing cultivation, harvest, slaughter, and so on. It is also essential for managing domestic resources in periods of resource scarcity when impacts that have not previously been experienced require adaptive strategies equal to the stress upon the agricultural system (Kennett and Marwan 2015).

NC research is primarily concerned with understanding why humans chose to ‘abandon more mobile [hunter-gatherer] strategies’ in favour of ‘well-defined resource catchment territories’ (Zeder 2015a, b: 3196). The transition from mobile hunter-gatherer communities to sedentary agriculture marks a stark transition in human societies’ cultural investment in fixed resources, infrastructure, and socio-political institutions (Zeder 2009). NC frameworks explain how, in various independent geographical contexts (cf. Smith 2011), resource-rich environments, communal living, and longevity collectively secured a predictable resource system predicated on selective plant and animal domestication (Zeder 2015a, b). But landscape modification and knowledge transmission cannot ensure adaptation to the local environmental conditions. Volatile climatic regimes and environmental degradation can undermine information transmission from past experience (Kennett and Marwan 2015) and in new environmental contexts (Rowley-Conwy and Layton 2011).

### Disequilibrium and Niche Construction

Research has recorded significant (often multi-decade to century-long) time delays between climate change and the response of vegetation communities (Svenning and Sandel 2013; Normand *et al.* 2013) that has challenged conventional models in community ecology that assume a uniform response to temperature change (Blonder *et al.* 2017; Svenning *et al.* 2015). Human communities can exhibit similar ‘adaptive lags’ when ‘the discrepancy between past and current environments […] produces a mismatch between behaviour and the environment’ (Laland and Brown 2011: 97).

As Riede (2009: 3) notes, ‘human tool-use and landscape modification are responsible for the […] hand-in-glove fit of human societies to their environment,’ yet even a highly flexible culture can ‘experience limits to its tolerance space, outside which it is unable to behave adaptively’ (Laland and Brown 2006: 98). This is because ecological changes operating outside collective experience of variation can limit the capacity of specialised strategies to yield predictable returns (Zeder 2016). For many complex societies, climate uncertainty has created disequilibria between specialised agricultural systems and the ecologies that are suitable to support them (Kennett and Marwan 2015). The pre-Hispanic Puebloan communities of the US Southwest illustrate how agricultural instability can result from the interplay between technological specialisation and unanticipated high-magnitude events. In the Hohokam, farmers overcame semi-arid environments by constructing a large-scale irrigation system supplied by the Lower Salt River (Howard 1993) that operated for over a millennium. However, the robustness of the system made it vulnerable to abnormal flow (Ingram 2008) and in the late fourteenth century, high flow devastated the system, causing regional depopulation (Nelson *et al.* 2012).

Agricultural expansion into new environments often signals the success of a particular mode of production, but can also lead to maladaptation. The Norse expansion across the North Atlantic islands in the Viking Age caused wide deforestation, vegetation clearance, soil erosion, and sometimes the extirpation of local species (Dugmore *et al.* 2005). The Norse showed a lasting resilience to feedbacks associated with environmental degradation. They were less resilient, however, to the impacts of climate variability and politico-economic changes in Europe (Dugmore *et al.* 2013).

### Adapting the Norse Economy in the Sub-Arctic

**Fig. 1:**
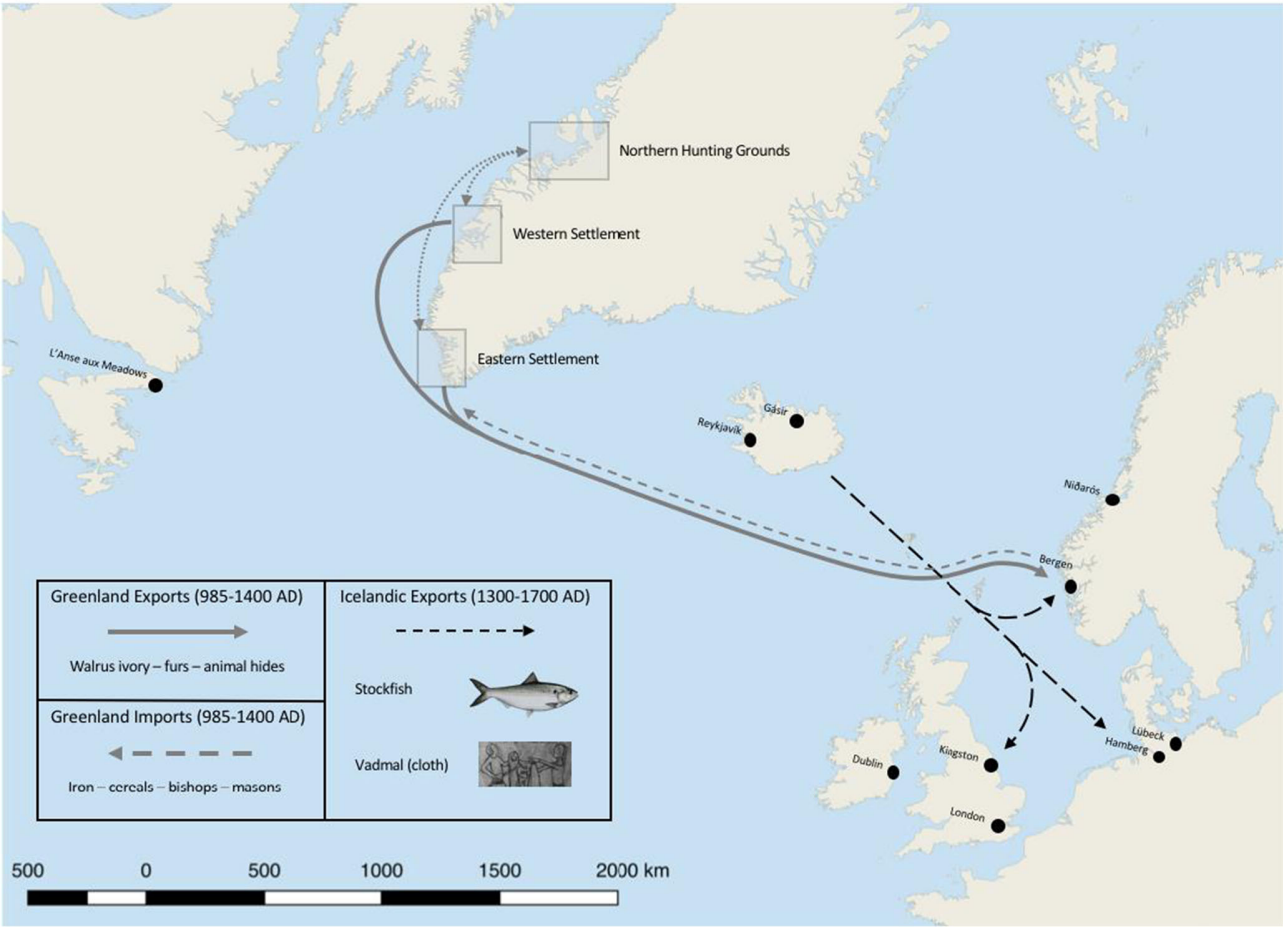
Showing the Norse Eastern and Western Settlement in Greenland and the Norse ivory and high-status goods export market between the late-10th and early-fifteenth century. Ivory was a high-value, low-bulk commodity used for lay and ecclesiastical adornments throughout Europe. In Greenland the largest concentrations of walrus were to be found in the Northern Hunting Grounds located at Disko Bay. Ivory would have then been refined in the settlements for export to Bergen on the royal vessel. The nature of export to Europe via Bergen represents a loose tie to wider European markets. Icelandic high-bulk, low-value goods conversely maintained diverse market ties to the German Hansa towns, England, Norway and Denmark

The settlement of Greenland (*c.* 985 AD) took place within a wider context of raiding, trading, and exploration known as the Viking Age (*c.* 793–1066 AD). Recent evidence suggests that the Norse settlers were seeking trade goods to replace ivory and hide lost following the extirpation of walrus in Iceland. In Greenland, walrus, fur bearing animals, and arctic exotica (i.e., narwhal) were abundant (Frei *et al.* 2015). Suitable lands for farming were located on the southwest coast in two main regions called the Eastern and Western Settlements (Fig. 1). The influence of oceanic and atmospheric circulation and seasonal sea-ice delivery result in average temperatures in Greenland that are ~8 degrees cooler than continental Europe at the same latitude (Dugmore *et al.* 2005). The inner-fjords of the Eastern and Western settlements support a largely continental climate, receiving less annual precipitation and lower average wind speeds than the more oceanic climate of the outer-fjords (Arneborg 2005).

Settlement patterns were characteristically dispersed and connected by networks of seasonally occupied shielings, bearing witness to the extensive utilisation of scattered grazing resources (Vésteinsson 2009). Zooarchaeological evidence indicates a *landnám* assemblage of domestic animals (cattle, caprines (sheep and goats), pigs, horses, dogs, and cats) characteristic of manor farms in contemporaneous Norway indicating the cultural significance of animal husbandry to Norse identity and status (Perdikaris and McGovern 2008). Subsistence strategies were modified to utilise local wild resources as the Norse switched from fishing and waterfowl supplements characteristic of Iceland and the Faroe Islands to hunt reindeer and seasonally abundant migratory seals (Smiarowski *et al.* 2017). Evidence of drive lines and hunting dogs to catch caribou (*Rangifer tarandus*) grazing near settlement areas suggests that Norse settlers imported terrestrial hunting techniques developed in Scandinavia (McGovern 1985). In addition, evidence of seabird and seal bones on inland farms indicates a network of exchange and a pooling of labour resources to maximise hunting efficiency. Techniques used in previous environments, such as communal boat drives, netting, and clubbing could have been used to harvest harp (*Phoca groenlandicus*) and hooded (*Cystophora cristata*) seals in the spring months (Dugmore *et al.* 2007a).

Early domestic assemblages were adjusted to suit long winters and constraints on fodder production (McGovern 1991). The quality and extent of pastureland and the availability of household labour would have determined the level of farmed food surplus possible (Vésteinsson *et al.* 2002). On small- and medium-sized farms, pigs disappeared and the proportion of cattle to caprine species declined (McGovern *et al.* 2014; Smiarowski *et al.* 2017). High-status farms appear to retain larger numbers of cattle (a principal symbol of social status). As survey data from Greenland’s Eastern settlement suggest, many low-status farms made radical transitions to hardier goat dominant livestock assemblages (Madsen 2014), however, low ratios of cattle were also maintained (McGovern 1994). Large livestock assemblages, the capacity to grow, harvest, and store large fodder reserves made elite farms more resilient to climate variability and extended periods of agricultural dearth. In similar periods, medium, and small farms would have relied on either supplementing production with wild resources or acquiring surplus production from large farms (Dugmore *et al.* 2012).

### The Seasonal Round: the Economic Year in Greenland

**Fig. 2:**
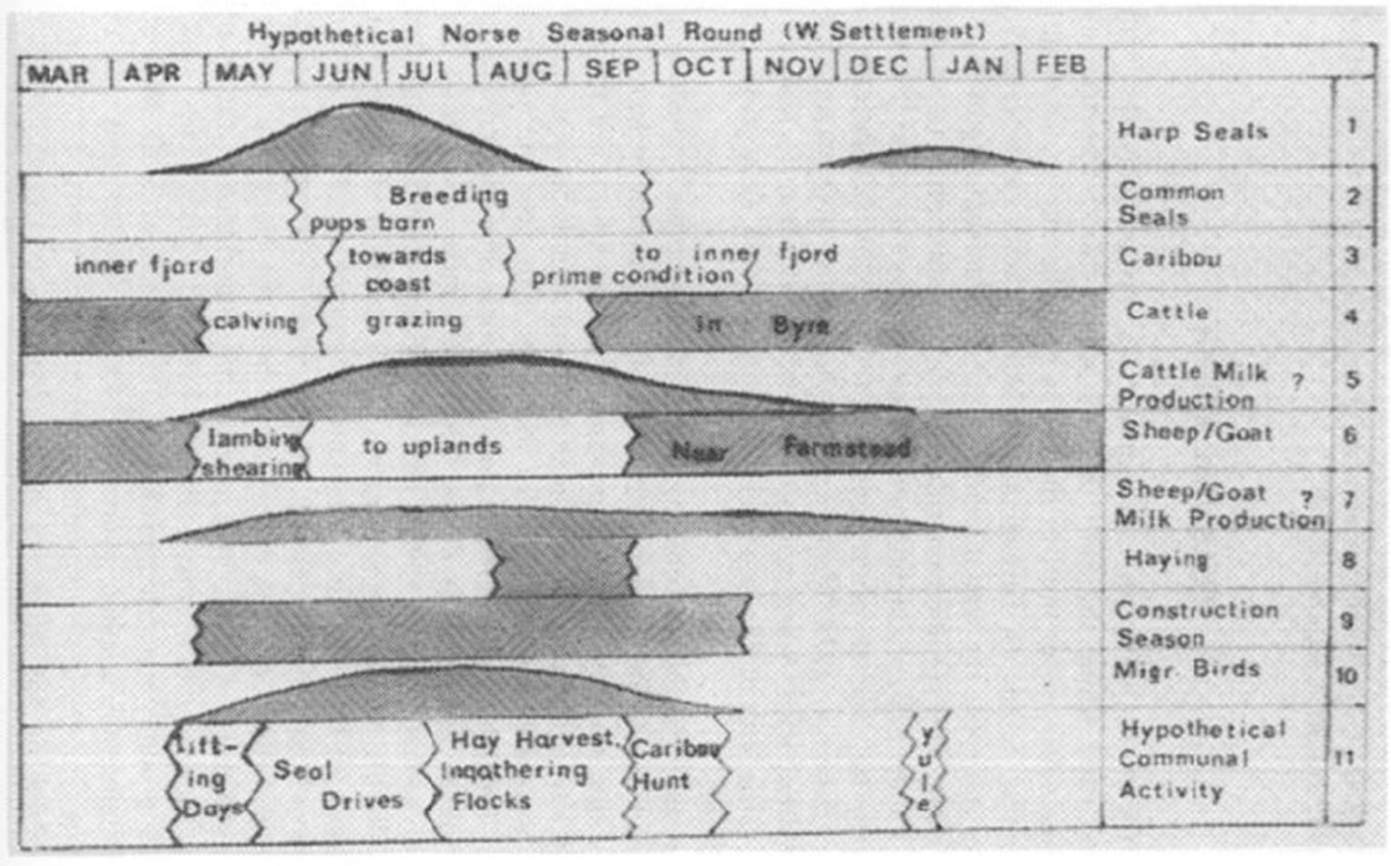
The seasonal round in Norse Greenland’s Western Settlement area. Grey shaded areas represent areas of high labour activity (after McGovern 1980)

The seasonal round of farming was organised with a notable concentration of activities and peak labour demand in the summer months (Fig. 2). In the winter months livestock were stalled in byres and grazed on stored fodder reserves harvested in the autumn months, and non-agricultural activities such as cloth production and the refinement of walrus tusks were carried out (Østergård 2004; Frei *et al.* 2015). In late spring, livestock were moved to the outfields and upland pastures to graze on the new growth; dairy production took place in specialised shielings (Ledger *et al.* 2013; Madsen 2014).

Core farming activities were balanced against additional hunting and foraging tasks across the wider land- and seascapes of southwest Greenland and long expeditions to the northern walrus hunting grounds (McGovern 1985; Enghoff 2003) and communal hunting drives to harvest migrating seals (McGovern *et al.* 1988). Zooarchaeological analyses have recorded an increased dietary reliance on migratory harp seals in both the Eastern and Western settlements from AD 1300 (Ogilvie *et al.* 2009; Smiarowski *et al.* 2017). Other communal hunting and foraging tasks would have taken place on a seasonal basis such as harvesting nesting birds in the spring and caribou hunting in the late autumn (Dugmore *et al.* 2007a).

Labour demand would have peaked between June and August, as hunters made the long and dangerous voyage to the Northern Hunting Grounds (*Nordurseta*) (McGovern 1985). Walrus ivory, hides, and other arctic exotica were high-value commodities on the European market, and were traded in return for essential resources such as iron (Gulløv 2008). *Grœnlandie vetus chorographia*, a seventeenth century text, describes the voyage to the *Nordurseta* as a journey that took six-oared boats between ‘15’ and ‘27’ days to reach the Disko Bay region (Frei *et al.* 2015). Heavy labour demands are likely to have coordinated a gendered division of tasks. In Norse societies, textile production was organised solely by women on the farmstead (Hayeur-Smith 2014). Runestone inscriptions and various written accounts from continental Scandinavia reflect the central role of women in farming tasks when men were away (Roesdahl 2016: 62).

Population estimates based on accounts in the *Vinland Saga* of the voyage to Greenland, ethnographic data from Iceland, and sustainable population models suggest a small peak around AD 1300. Assuming an initial population of 300–500 individuals, and an exponential growth rate of 0.62%, population is most likely to have peaked at ~1400–2200 by AD 1300 (Lynnerup 2014). A population of this size divided between the Eastern and Western settlements would have made labour management difficult in the summer months. Although absolute population numbers remain uncertain, there is good evidence for exceptional labour demands. Skeletal remains indicate both hard labour and a regular use of teeth as tools (Scott *et al.* 1992; Scott and Jolie 2008). Despite a protein-rich diet and limited exposure to the diseases endemic in contemporary Europe, life was likely to have been brutally hard for most of the Norse settlers even in periods of comparative prosperity.

### Exogenous Change

Archaeological evidence suggests that, from the mid thirteenth century the Eastern settlement became increasingly vulnerable to higher-scale changes across the North Atlantic. Increased climate volatility, changes to market structures and trading systems in continental Europe, and the possibility of hostility with the Thule Inuit culture have gained significant attention in multidisciplinary studies over the last 40–50 years (McGovern1994; Dugmore *et al.* 2007a; Nelson *et al.* 2016).

### Climate Change

Despite the relative mildness of the Medieval Climate Anomaly (MCA) even early settlers would have experienced harsh winters and summer droughts in Greenland (Dugmore *et al.* 2007b). Nevertheless, the Norse proved highly adaptive, establishing and expanding settlement range until the twelfth century (Madsen 2014). From the mid-thirteenth and fourteenth centuries multi-decade cooling increased the length and harshness of winters, sea-level rise submerged coastal home-fields, and storminess and summer sea-ice increased (Kuijpers *et al.* 2014; McGovern *et al.* 2014).

**Fig. 3:**
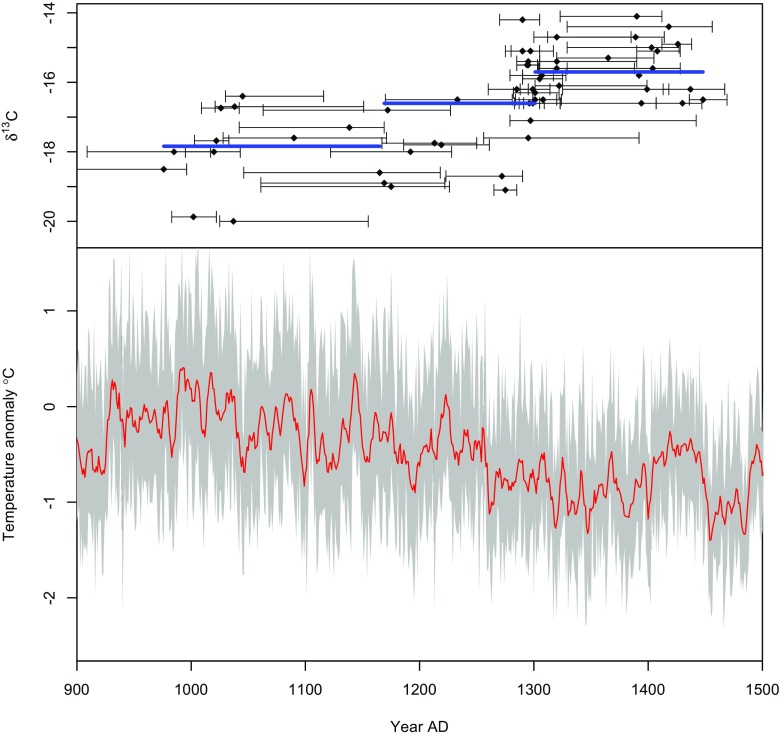
Isotope records (above) collected from human skeletal remains in Norse Greenland indicating a transition from high terrestrial low marine to low terrestrial high marine with radiocarbon error bars and median lines (Arneborg *et al.* 2012). Multiproxy temperature records (below) for the Arctic-Sub-arctic region (60–90^°^N) between 900 AD and 1500 AD (McKay and Kaufman 2014). Thick line indicates 5 year moving average to show the multicentury cooling trend commencing from the mid-thirteenth century. There is a negative correlation between temperature anomaly and isotopic signature indicating increasing adoption of marine proteins as the climate cooled

Recent climate models suggest that North Atlantic cooling was triggered by a series of globally significant volcanic eruptions in the thirteenth century, culminating in the eruption of the Mt. Samalas Volcano in AD 1257–1258; each event reducing solar insolation and increasing the onset of summer sea-ice in the Denmark Strait (Miller *et al.* 2012; Lavigne *et al.* 2013). The onset of the generally cooler and more variable Little Ice Age (LIA) stressed farming economies; homefields became less productive and longer winters increased livestock mortality, causing a decline in dairy production (McGovern *et al.* 2014). Results from isotopic studies of human bone collagen indicate a transition from a largely terrestrial diet supplemented by ~40% marine resources in the eleventh century to a diet topping ~80% marine input in the final phases of settlement (Arneborg *et al.* 2012) that coincides with climate cooling commencing in the mid-thirteenth century (Fig. 3). Hydrographic proxies also record cooling in the fjord systems of southwest Greenland (Jensen *et al.* 2004; Kuijpers *et al.* 2014) and increased summer sea-ice prevalence, which would have affected boat travel, seal migration routes, summer grazing patterns, and fodder availability across the Eastern and Western settlements (Ogilvie *et al.* 2009; McGovern *et al.* 2014).

In the Eastern Settlement consumption of non-migratory harbour seals (*Phoca vitulina*) declined significantly after AD 1300 likely due to sea-ice, displaced harbour seal populations, and increasing dependence on migratory seal species (Ogilvie *et al.* 2009). Ecological changes such as these would have stressed the application of TEK and increased the ‘degree of adjustment’ required to utilise coastal resource spaces effectively (Berkes 2017).

### European Economic Transition

In continental Scandinavia, the impact of the Black Death (AD 1346–48) in Norway and the Kalmar Union of 1397 AD effectively shifted the center of power from Norway to Denmark (Epstein 2009). Danish royal and mercantile interests were focused upon the Baltic and North Seas, with increasing trade with Russia opening-up sources of walrus products in the Barents Sea by 1400 AD. In Europe by the fourteenth century, power and trade had accumulated around a coalition of northern German towns, shifting Scandinavian societies to the periphery of the European world-system (Nedkvitne 2014).

These transitions on the European continent had contrasting impacts on North Atlantic trade. Unlike Iceland, Greenland lacked the combination of a laboring population, grazing sheep herds, and access to fish stocks to generate surpluses of wool and dried fish for export (Madsen 2014). An absence of suitable ship-building timber in Greenland limited the possibility of local production of large ocean-going boats, making the Norse solely dependent on visiting boats from Europe. With waning Scandinavian royal interests in its distant Atlantic outpost, stiff competition from other suppliers of arctic products (i.e., Sami and Karelian trades), and lack of dried fish or woolen goods to attract commercial interest from the Hanseatic League (whose cog ships were ill-suited to the stormy North Atlantic), the Greenlanders were unable to maintain economic ties with Bergen leading to increased isolation from the early fifteenth century (Dugmore *et al.* 2007a).

### Cultural Contact

Little is known of the possible interactions between either the Dorset Paleo-Eskimo or the later Thule Inuit and Greenland Norse, and hostilities mentioned in the *Vinland Sagas* and Ivar Bardarson’s accounts remain ambiguous and uncorroborated in the archaeological record (Appelt and Gulløv 2009). After 1300 AD, a written record from the Icelandic Annals suggests at least intermittent hostility with small groups of Norse men and boys reported killed or carried off, perhaps during voyages northwards (Gulløv 2008). However, there is limited evidence in the archaeological record to corroborate such accounts. A recent large-scale genetic study of the modern Greenlandic population found no evidence for any admixture with Norse or Dorset populations and argues for a single, substantial migration event (Moltke *et al.* 2015). Hostility and estrangement of Inuit cultures is likely to have reduced the possibility of inter-cultural transmission of ecological knowledge.

## Discussion

### Cultural-Ecological Disequilibria

Preindustrial agrarian states were dependent on predictable climatic conditions for planning utilisation of local-scale resources (Kennett and Marwan 2015) and on accumulated information about the opportunities and limitations of the environment and the capacity of social systems to utilise resources efficiently (Riede 2011, 2012). In farming communities, resources diversification, storage, yield-boosting technologies and social-economic institutions can be used to increase the predictability of local resources (Zeder 2016). But when communities have insufficient knowledge to organise sustainable responses to changing social-ecological feedbacks, the system can become vulnerable to food shortages (Nelson *et al.* 2016). In many preindustrial societies climate volatility or unanticipated climatic events (i.e., floods or drought) undermined centralised political authority leading to decentralisation and diverse adaptive pathways (Kennett and Marwan 2015; Middleton 2017). This was often associated with path dependent institutions, structures, or behaviours that resulted from historical antecedents of cultural adaptation and environmental change.

In Norse Greenland, the transition to less predictable climate conditions resulted in resource diversification (Smiarowski *et al.* 2017), the use of storage and irrigation structures (Buckland *et al.* 2009), and manuring and soil augmentation (Adderley and Simpson 2006) to maintain population stability. Acute climate uncertainty in the fourteenth and fifteenth centuries did not, however, result in reduced political complexity—as in many cases of societal transformation (Tainter 1988; Butzer 2012). In fact, evidence of church building in the fourteenth century suggests a strengthening of politico-religious institutions (Arneborg 2003). Adaptive strategies were largely uniform across the settlement (with the exception of differences in household-scale diet) and aimed at sustaining farming (Dugmore *et al.* 2013). As we argue, it was a combination of historical-cultural factors and changes operating across different spatial and temporal scales that caused social-ecological disequilibria in Norse Greenland.

### Antecedent Landscapes of Norse Greenland – Niche-Construction in Scandinavia

**Fig. 4:**
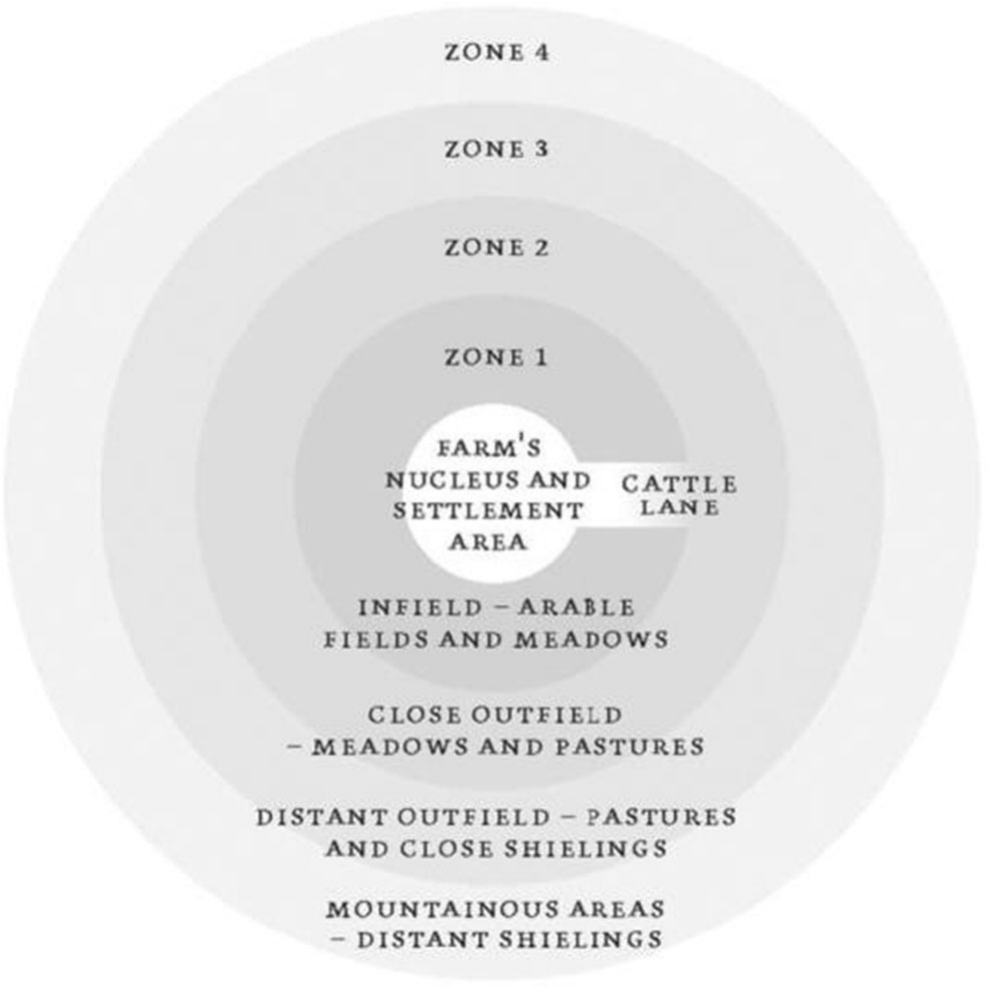
Conceptual model of the Norwegian farm and legally regulated areas (after Øye 2013)

The arrival of agriculture and domestic animals to Scandinavia was part of a complex process of cultural interactions and extension (Rowley-Conwy 2013). From as early as the Late Bronze Age, farm institutions were formed, establishing a spatial continuity for organising the household-barn and farm structure (Myhre 2004). This created conformity to the spatial organisation of the farm and seasonal practices of agriculture in much of Norway, and institutionalised and embedded ideas and practices of niche modification (Øye 2013). By the Medieval period, farms were comprised of a central nucleated cluster of buildings, field systems, and shielings (Fig. 4). Seasonal transhumance was organised between winter byres and close pasturelands and more distant summer pastures, as the cultural niche was expanded into broader the landscape. Differences in climate across Norway, however, dictated the amount of cultivation, stocking capacity, and wild resource use that was possible (Øye 2004).

The transformation of Norway’s natural environment into a network of cultural landscapes and the evolution of integrated agricultural systems, technologies, and institutions by the Middle Ages (Øye 2004) left a “legacy of modified selection pressures” operating over millennia (Laland *et al.* 1999: 10242). The ‘ecological inheritance’ left by landscape modification processes, which included vegetation clearance and burning, soil augmentation and manuring, irrigation, and managed grazing (Mehl and Hjelle 2017) produced anthropogenic soils and ‘cultural steppe’ landscapes for descendent populations (Dugmore *et al.* 2005, 2006). This increased predictable returns in the farming niche (Zeder 2016). Technologies and practical knowledge had evolved to effectively manage crop and hay fodder production, seasonal transhumance, and specialised hunting strategies (Perdikaris 1999). Axes, ploughs, tillage and harrowing tools, scythes, and storage technologies were used to intensify local-scale production and store resources through periods low productivity (Øye 2009). Knowledge of local resources and agricultural skills would have been transmitted and updated through social learning, stories, and beliefs leaving an ecological and cultural legacy in Norway that was resilient to the long-term constraints of the environment (Øye 2004; Riede 2012).

The accumulation of knowledge informs expectations of resource acquisition long before they are encountered and so colonisation could have presented an acute challenge to knowledge and practices established in homelands as they mismatched with the environments of new settlements (Rowley-Conwy and Layton 2011). As a result, evolved cultural-ecological (or biocultural) behaviours need to be considered as structures of adaptive knowledge in the North Atlantic.

### Contextualising the Norse Cultural Niche in the North Atlantic

Climate-adaptation can be understood through two macroevolutionary processes that allow cultures to become adapted to their environments. The first—*landscape learning*—explains the exploratory processes whereby individuals and groups gather and share information about the locations, limitations (resource fluctuations and interannual weather extremes), and the organisation of resources within a local catchment (Rockman 2003). The second process—*cultural transmission*—is closely interwoven with landscape learning. Information is accumulated through observation of local resource systems and recombined into the existing adaptive package (Boyd *et al.* 2011). Culture plays a critical role in accumulating, transmitting and, at times, limiting human adaptive capacities in new environments.

### Accumulating Information – Landscape Learning in Greenland

It has been proposed that early ‘hunters and scouts’ would have explored and assessed the resources in Greenland before the initial settlement in 985 AD (Dugmore *et al.* 2007a). The abundance of valuable walrus colonies and marine resources within reach of the settlement areas would have required a scoping phase to assess whether a farming niche could be established (Dugmore *et al.* 2007a; Frei *et al.* 2015). Suitable landscapes for animal husbandry practices were located in southwest Greenland. Climate data indicate that settlement took place in the comparatively mild and stable conditions of the MCA (Dugmore *et al.* 2007b; Nelson *et al.* 2016). In the early phase of settlement, stable climatic conditions would have increased the predictability of selective returns from hay cultivation and the cycles of livestock transhumance. Knowledge of climate variability and its effect on local ecosystems would have been accumulated over the initial settlement period. This allowed existing economic activities to be adjusted to the limitations of the environment. The stable climate helped early settlers predict ecological feedbacks that influence the timing and location of plant growth, marine, and terrestrial mammal migration and domestic livestock reproduction (McGovern 1980).

In agricultural economies familiarity with feedbacks are manifest as economic cues and seasonal structures of labour organisation (Kennett and Marwan 2015). The identification of local resources allowed environmental information to be reincorporated into existing organisational structures—the Norse seasonal round (McGovern 1980; Fig. 2)—by adjusting existing practices, but critically in ways that conformed to the identity of the existing Norse ‘cultural niche.’ The limited availability of standing wood for the construction of longhouse structures, for example, meant wood beam supports were replaced with perpendicular internal walls to support the roof (Høegsberg 2014). Further research, including comprehensive survey work in the Vatnahverfi region of the Eastern Settlement, has recorded a general absence of infield dykes, which are common to contemporaneous Norwegian and Icelandic farms, to separate grazing livestock from cultivated home-fields (Madsen 2014). This absence, combined with evidence of intensive use of shielings, suggests a less intensive type of farming that utilised broader grazing resources (Madsen 2014). Lower primary production in Greenland would have made this a necessary strategy to reduce the chance of erosion on outfield pastureland. Zooarchaeological evidence indicates a gradual adjustment of livestock ratios to favour species that were suited to the longer, harsher winter conditions. This corroborates with isotopic data, indicating an increased reliance on marine mammals, as well as a continuing reliance on caribou hunting (Arneborg *et al.* 2012; Smiarowski *et al.* 2017).

By accumulating information about the location and limitations of the environment in southwest Greenland, the Norse were able to suitably adjust their existing agricultural niche to the new resource regime. However, accumulated information in this early phase of settlement merely retrofitted the existing Norse ‘cultural niche’ to conform to mild and predictable conditions – precluding larger-scale adjustments that buffer against environmental stress. In other words, the Norse agricultural system was able to operate under the assumption that resources would remain located in specific places and times within the economic year (McGovern 1994). Because farming societies are information-intensive uncertain conditions can reduce farmers’ capacity to “evaluate the costs and benefits of one strategy or another” (Kennett and Marwan 2015: 2–3). Buffering strategies used in past environments, for example, are unlikely to have operated effectively as the climate deteriorated.

Home-field management was fundamental to temperate European pastoral economies, and irrigation and manuring strategies were widely employed to increase productivity—often to counter declining home-field yields. These imported management strategies could sustain fodder production through cold winters and summer droughts that were characteristic of MCA conditions in Greenland (Dugmore *et al.* 2007a, 2007b). Evidence of irrigation and manuring is widespread across the Eastern (i.e., Gardar) and Western settlements (Buckland *et al.* 2009), but the productiveness of this strategy would have declined as multi-decadal cooling enhanced positive feedbacks reducing home-field yields (Golding *et al.* 2015). In milder climates manuring can boost soil fertility (and yield) but in Greenland an increase of organic material within the soils enhances water retention leading to a build-up of winter ground-ice and a delayed spring thaw, shortening the effective growing season (Adderley and Simpson 2006). Prolonged winters and decreased fodder production would have increased livestock mortality, reinforcing dependence on hunting and foraging strategies to support subsistence (Dugmore *et al.* 2012). Unanticipated feedbacks such as these would have required the Norse to respond by stretching the application of short-term buffering strategies with the effect of reducing the size of their ‘safe operating space.’

### Cultural Disequilibria – Norse Agriculture and Thule Hunting

To understand the level of disequilibrium between the Norse ‘cultural niche’ and the environment, it is necessary to consider how the Norse adaptive package was suited to long-term ecological change in sub-Arctic Greenland, and this can be achieved through a comparison of the Norse and Thule Inuit cultural niches. Thule society, direct ancestors to modern-day Inuit, evolved a highly adaptive ‘cultural niche’ comprising habitat-specific knowledge, complex technologies, and behaviours required to survive in the extreme cold (Gulløv 2008). Archaeological and ethnographic sources record highly varied material cultures and practices comprising different bows, arrows, and harpoon technologies, styles of clothing, and designs of kayak (Park 2005; Mason 2007). This technological assemblage had evolved over millennial timescales, as knowledge of resource-use was accumulated and recombined (Boyd *et al.* 2011).

The Norse ‘cultural niche’ differentiated between a socialised ‘inside’ of fixed dwellings and modified cultural landscapes and a wild, chaotic ‘outside’ (Arneborg 1997; Gulløv 2008). Modified landscapes conformed to European agricultural economies: local intensification, economic division and cyclical organisation of field systems, domesticated animals and human labour (McGovern 2000; Golding *et al.* 2015). Technologies were highly evolved to support the European farming niche. Axes, scythes, sickles, spades, soapstone storage vessels, and cooking pots are found from the beginning to the end of settlement (Arneborg 2000; Enghoff 2003). Clothing manufacture also corresponded with Medieval European styles and production techniques, with minor differences to design, and would have utilised wool from domestic sheep and goats (Østergård 2004; Hayeur-Smith 2014). These technologies and practices starkly contrasted with the Thule Inuit ‘cultural niche’ (Table 1). Beliefs, practices, and technologies were integrated with the Arctic environment in the Inuit lifeworld—connecting humans and animals, even in the act of hunting, killing, and consuming animal flesh for food and skins for clothing (Ingold 2000). In Inuit cosmologies, the exchange of vital forces between humans and animals is reciprocal, requiring a deep knowledge of animal behaviours and biology (Brody 2001; Berkes 2017). Technologies were also highly evolved: caribou skins were harvested to construct highly insulated and pliable shelters, parkas, stockings, and boots (Meaks and Cartwright 2005).

**Table 1:** Norse and Inuit Cultural Niches

	Norse	Inuit
World view	The Norse socialised dwelling and cultural landscapes constructed a differentiation between a socialised “inside” and the chaotic and untamed “outside” of the natural world. From *c.* 1000 AD, after the Christianisation of Norway and Iceland, Christianity became the core belief system in Greenland—as reflected in institutions, art and social organisation and structures (Arneborg 1997; Gulløv 2008).	The Inuit animist cosmology situates the self within and in reciprocity with nature. Existence is suspended in a flow of vital force between humans and nature. Hunting influences the circulation of vital forces between animals and humans and contributes to the ‘regeneration of the lifeworld of which both are part’ (Ingold 2000: 114). The animic understanding of the world should be considered a dialogue and exchange – with it delivering a depth of habitat-specific knowledge.
Technology	Technologies evolved to increase the efficiency and predictability of domesticated plant and animal resources. Structures and associated legal codes demarcated land rights. Storage vessels, metal tools, institutional structures, and clothing were all produced, using local production or trade. The Norse (and Celts) also utilised local wild resources peripheral to the farm (Jesch 2015).	Clothing, hunting bows, arrows, spears and harpoons, knives and kayaks and dog sleds were interwoven with local ecology and the Inuit world view (Park 2005). Technologies were highly-evolved, using local resources to accumulate and innovate material design for efficient use (Boyd *et al.* 2011).
Social Organisation	Landscapes were modified to accommodate farm animals and cultivated hay meadows for winter fodder. Storage was required and therein a fixed mode of social organisation, that relied on prediction and seasonal timing of cycles of growth for agricultural returns. Inter-farm networks supported the circulation of materials, labour and livestock to support survival (Arneborg 2008; Buckland 2008).	Small, nomadic communities of closely related hunter-gatherers. Settlements were temporary and seasonal, and would involve mobile hunting, cache storage and intermittent trade with other Inuit and ethnic groups (Gullov 2004, 2008).
Ecological Knowledge	In situ *–* Local knowledge gained from short-term *landscape learning* to locate, observe and manage ecological cycles in southwest Greenland (Rockman 2009). Ex situ *–* Imported Norwegian-Icelandic mixed farming practices supported local-scale production using irrigation and manuring to intensify yields (Dugmore *et al.* 2007a, 2012).	Ex situ – High-Arctic Canada. Seal hunting on ice and in water using knowledge of ice conditions to navigate safely and kayak technologies to operate in open water. In situ – Local knowledge is gained from the continuity between high-Arctic and sub-Arctic ecologies. The Thule Inuit advanced southwards from across Elsmere island from the 12th–13th centuries.

Norse and Thule Inuit cultural niches supported highly different behaviours. The Norse constructed a cultural niche to support local intensification, while Inuit lifeways observed intimate details of extensive Arctic habitats. Archaeological and genetic evidence, however, suggests contact between the Thule Inuit and the Norse Greenlanders (Gulløv 2008; Raghavan *et al.* 2014). By the fourteenth century, Thule culture had advanced to the outer-fjords of the Norse settlement areas, most likely for summer trading (Golding *et al.* 2011). Evidence for exchange of Norse (i.e., chessman – Neo-Eskimo Ruin Island phase) and Thule Inuit (i.e., Neo-Eskimo bird, hunting implement – Norse Eastern Settlement) artefacts has been recovered from high-Arctic Thule and sub-Arctic Norse contexts (Gulløv 2008). But evidence of cultural contact and exchange does not mean ‘social learning’ was possible between the Norse and Inuit cultures. As Gulløv (2008) explains, chess never existed in prehistoric Arctic cultures, and harpoons were never adopted in Norse society; they may have conveyed a (new) symbolic value but use-value is improbable without a compatible knowledge of the other culture.

The Norse and Inuit had vastly different social arrangements and settings for ‘social learning’ practices (Arneborg 1997). This is because the “acquisition and retention of beliefs, values, role expectations and skills” were situated in different cultural-ecological contexts (Kendal 2011: 241). Because the Norse failed to abandon agricultural practices, it is likely that social learning was situated on the farm (McGovern 1991). Cultural practices were essential to the transmission of information vital for enhancing returns from selected livestock. Miniatures of farm animals, swords, and boats are found on Norse sites across Scandinavia and the North Atlantic (Arneborg 2000; Gardela 2012). These can be interpreted as ‘qualifier toys’ that deliver a common sense of identity and operate as a primer for agricultural practices in later life (Riede *et al.* 2018). In late childhood, children would have undertaken shepherding duties and assisted with other tasks on the farm (Dugmore *et al.* 2007a). Intergenerational cultural transmission would have delivered accumulated information about local environments and how the Norse ‘cultural niche’ fits within it (Riede 2012). But because learning was situated within the Norse ‘cultural niche’ it is unlikely that innovative information about the wider environment beyond cultural landscapes was easy to acquire and assimilate with existing behaviours.

Social learning in the Thule ‘cultural niche’ is understood through a well-preserved material culture and contiguous ethnographical records (Park 2005; Riede *et al.* 2018). Ethnographic research in the early twentieth century records children learning complex practices of seal and caribou hunting through play using miniature bows and arrows, harpoons, and kayaks (Park 2005) and carved miniature animal figurines that draw attention to animal behaviours and physiological features. This allowed the accumulation of knowledge about Arctic environments, animal behaviour, and technology craft and use—and technologies could be fine-tuned (“ratcheted-up”) to environmental variations, increasing efficacy (Riede *et al.* 2018). The level of complexity and technological know-how observed in the Inuit ‘cultural niche’ is the result of the long-term accumulation of adaptive elements to construct an adaptive package for the dynamic Arctic environments of Greenland and Northern Canada.

Critically, while there is evidence of Norse-Inuit artefact exchange, there no evidence of inter-cultural transmission of adaptive behaviours or learning practices. The Norse adopted incremental changes within their existing ‘cultural niche’ (Table 2) but did not adopt new behaviours. Changes to Norse material culture include increasing the thread-count of clothing (Hayeur-Smith 2014), increasing dependence on hunting marine mammals (Arneborg *et al.* 2012), and adjustments to farmsteads, field systems, and shieling use (Madsen 2014). This strongly suggests a ‘conformist transmission’ of cultural information (Laland and Brown 2011), maintaining the resilience of the Norse agricultural niche at the expense of efficient resource-use in the long-term. The conformity of behaviour is unsurprising as cultural evolution takes place over long periods of time (Bentley and O’Brien 2015) and is reinforced by the institutional setting—which, for the Norse, included local governing (lawmakers and chieftains) and religious (parish churches) institutions (Vésteinsson 2009).

**Table 2:** Incremental adjustments made to Norse 'Cultural Niche' - an overview of the North Atlantic

Technology/Structure	Greenland	Iceland	West Norway	References
Cloth	Continuity in artisanal (household-scale) cloth production. Evidence of recycling and incorporation of additional animal fibres into to clothing, including goat hair. Concentration of wool fibres are indicative of adaptation to cooler summer/winter conditions. No evidence of Inuit-style seal-skin clothing in Norse sites.	From 14th and 15th centuries AD, cloth production transitions from artisanal to legally regulated production (measured in ells) for exchange and sale on European export markets. The Grágás legal codes record detailed disputes over exchange of vadmal.	Textile production becomes ostensible in Viking Age burial customs. Clothing and other material fragments demarcate social status and gender in inhumed burials. Wool combs, spindle whorls, loom weights, weaving beaters, and shears have been uncovered from Viking Age burials in west Norway – indicating the important role of textile production in Norse society.	Hayeur-Smith, 2013; Hayeur-Smith *et al.*, 2016; McGovern 1994; Østergård 2004, 2011; Øye 2013
Iron and Tools	Iron tools are found across the Eastern and Western settlements. As in Iceland and west Norway, these tools were essential for farming tasks, including vegetation clearance, hay cultivation, maintenance and hunting. On some farms tools, such as axe-heads and belt buckles, have been uncovered, forged from whalebone and walrus ivory because iron became less readily available as imports declined.	Though Iceland was limited by available wood, there is extensive evidence of charcoal production for extracting iron from iron ore. Metal working has persisted over the last 1100 years in Iceland. Iron tools used for farming and warfare (see Norway) are found across Icelandic assemblages.	Iron production grew rapidly in the early Medieval period, increasing the efficiency of farming equipment by sheathing working parts. Cultivation, harvesting and expansion of farmland were enhanced by axes, picks, sickles, scythes and spades.	Arneborg 2003; Seaver 1996; McGovern 1980, 1985; Kopár 2009; Øye 2004; Dugmore *et al.* 2006; Church *et al.* 2007
Wood	Limited forest cover. Dwarf willow (*Salix sp.*) and birch (*Betulia nana* L.) scrub predominates in the Eastern and Western settlement areas. Significant use of driftwood to construct and repair tools, boats and built structures. Religious icons would have been carved from wood, but the Norse also carved bone and ivory figures.	Pre-landnám lowlands and interior highlands predominantly dwarf birch, willow and juniper scrub. A significant number of wood artefacts are found on high status farms in Iceland. Wood resources were highly valuable for iron smelting and standard farm tools and structures including axes, scythes, fencing and housing structures.	Wood was one of the most important materials in Scandinavian societies. It was used in building supports, boats, farm tools, fencing structures and ecclesiastical adornments. Wood provided essential tools for modifying environments, especially in composite form with iron sheathings.	Høegsberg 2014; Dugmore *et al.* 2005, 2006; Øye 2004; Myhre 2004
Shielings	Shielings had broader functions that west Norwegian examples. A combination of outfield tasks could be organised in these structures. Such tasks included transhumance, interfirm exchange and bases for hunting terrestrial and marine resources. The absence of infield dykes on many farms suggests that shielings also played an important role keeping animals away from cultivated hayfields.	Shielings are similar in characteristic to the tripartite division of shielings described in Reinton’s (1960) model. Shieling areas were usually located on the outfields and at a moderate altitude on summer pasturelands. Shielings were usually used as milking stations and to manage upland grazing.	Ethnographic and historical data collected by Reinton (1960) describe a tripartite division of shielings into 3 categories: *dairy shielings* – used for near-farm milk production – *haymaking shielings* – used for the collection of hay-fodder for the winter – and *full shielings* – with combined functions, sometimes including summer and occasional winter residence.	Madsen 2014; Keller 1989; Reinton 1960; Sveinbjarnardóttir 1991; Øye 2009
Boats	Few large vessels (Knarr and Longboat) would be available to the Norse Greenlanders because of limited timber resources. Driftwood would have been plentiful and iron bearings were imported from continental Scandinavia. Six-oared are recorded in *Grœnlandie vetus chorographia* as transportation for voyages to walrus hunting grounds and would have been used in the spring seal hunts to drive seals ashore.	The absence of standing timber (as in Greenland) would have made shipbuilding difficult. However, the Norse maintained a close connection with Scandinavian homelands, and frequent journeys were made between Iceland, Norway and the Northern British Isles.	By the Viking Age, the west Norwegian Scandinavians had developed a highly specialised maritime culture. Longboats had evolved agile manoeuvrability through waves, aided by the clinker design, and a large protractible square sail allowed speed over the open seas. This was a keystone of the raids on the British Isles and the Low Countries, but also formed the basis for long-distance trade and settlement in the North Atlantic. The appearance of ships on rock art, coins and other forms of art, as well as its significance in burial rituals made the ship highly significant to the Norse.	Dugmore *et al.* 2010; McGovern 1980, 1985, 1994; Frei *et al.* 2015; Bill 2008; Barnett 2015
Farm (Farmstead and Outbuildings)	Longhouse and passage house designs have a strong continuity with Icelandic internal spatial arrangements. Differences are associated with use of local materials on account of limited standing timber. In his extensive household survey, Roussell (1941) observed a 'centralized farm' type--an adaptation that minimised the use of wood and turf, while the clustered layout maximised heat conservation over the long winter months. Høegsberg suggests this spatial continuity of longhouses to be indicative of ‘diasporic regionality’.	Longhouses hold a strong continuity with the standard Scandinavian model. Houses would have consisted of between one and five sunken-featured buildings and a long hall with a central hearth and a timber frame and supports. Limited standing timber led many longhouses to innovate, using local stone and turf resources rather than wood beam roof supports. The use of turf walls and stone to partition rooms is more common – showing a conformity with Greenland.	In the late Neolithic Period (2400–1700 cal. BC) Southern Scandinavian style (similar to contemporaneous Danish) longhouses made from wattle and daub were constructed in western Norway. By 1500 BC the ‘three-isled longhouse’ composed of three longitudinal rooms supported by two parallel rows of beams. In the early Bronze age, buildings divided into living quarters and barns were developed.	Høegsberg 2014; Smith 1995; Madsen 2014; Myhre 2004; Vesteinsson 2006; Roussell, 1941
Field Systems	Extensive survey work in the Vatnahverfi region of the Eastern Settlement, infield dykes are less common than in Iceland and Norway. This indicates a less intensive type of farming. Though this could be explained by smaller livestock herds and intensive use of shielings for herding.	The Icelandic field systems conformed with Norwegian, with the addition of complex resource rights beyond the farm infield and close outfields. This is partly down to the complex geomorphology of Iceland. Certain farms had access rights to mountain pastures far from clustered farm buildings.	Farm buildings (*farmstead, and livestock and storage buildings*) were located within the infield area, which was in turn demarcated from the outfield by a dyke. The dyke prevented livestock from moving between the outfield pastureland and cultivated hayfields.	Øye 2004, 2009, 2013; Sveinbjarnardóttir 1991; Dugmore *et al.*, 2006; Madsen 2014;

### Hierarchy and Conformist Adaptations

Collective behaviours are likely to conform to successful or trusted institutions (Boyd *et al.* 2011; Thompson 2013). The Grágás and Jónsbók law books record legal codes on resource access, governance, and disputes in Iceland, but also likely reflect modes of governance and organisation in Norse Greenland (Sandvik and Sigurdsson 2005). These codes would have regulated the exchange of goods, resource access rights, and the organisation of agricultural land (Madsen 2014). Settlement data also indicate a hierarchical structure. The transition from small annex church structures of the eleventh century to centralised parish churches between the twelfth and early-fourteenth centuries, suggests a two-tier division of society between a church-lord class controlling rich pasturelands with surplus production capacity and a homogenous lower-class struggling to maintain farm production (Vésteinsson 2009).

Differences in private resource access would have reinforced the dependence of lower class farms on larger farms that controlled the distribution of resources (Dugmore *et al.* 2012). As Thompson (2013) explains, hierarchical institutions are likely to assimilate the impacts of environmental change to stabilise safe limits. By controlling the redistribution of surplus resources to smaller farms, elite farms could maintain a uniform dependence on standard modes of production, limiting capacity to diversify adaptive strategies.

### Adaptive Strategies Created New Vulnerabilities – Changing Settlement Patterns

**Fig. 5:**
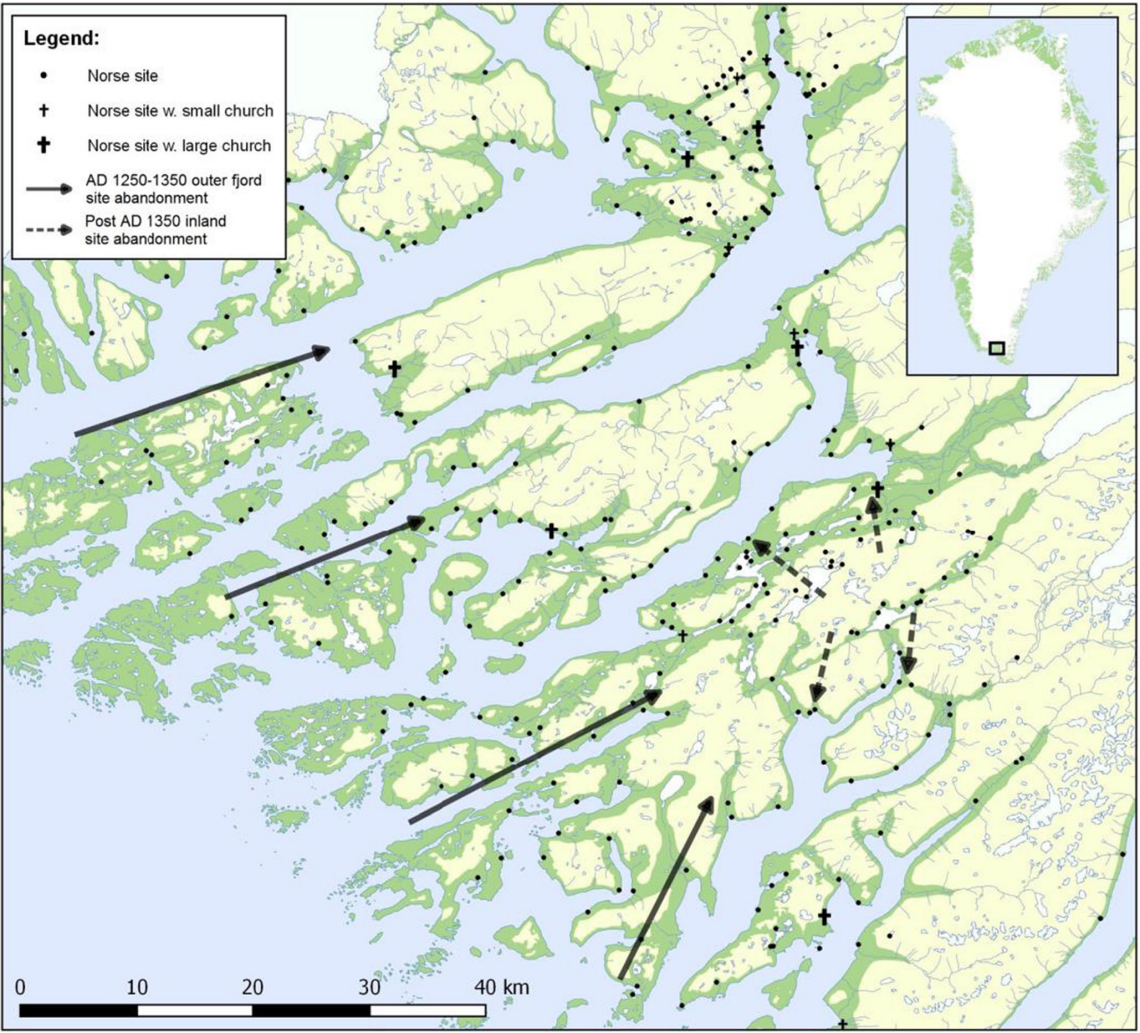
Abandonment of outer-fjord and upland areas of Norse Eastern Settlement (cf. Madsen 2014)

Relocation has long been considered an adaptive response to risk but can also produce unforeseen risks and trade-offs (Halstead and O'Shea 1989). Changing environmental conditions from the mid-thirteenth century are thought to have created significant vulnerabilities among farms established in the late-tenth and eleventh century settlement phase (McGovern 1994). Survey data collected from Greenland’s Eastern settlement suggests a reorganisation of settlement from distinctly dispersed patterns across the inner and outer fjords to more concentrated settlement on the inner fjords from the thirteenth century (Vésteinsson 2009; Madsen 2014). Farm abandonment on outer fjord and upland environments likely reflects changing environmental gradients as pastureland became less productive for harvesting winter fodder (Madsen 2014). Recent palynological studies suggest decreased grazing pressure and abandonment of Saqqaa (*c.* AD 1350–1400), Lake Vatnahverfi (*c.* AD 1290–1400), and Saqqaa Tasia (*c.* AD 1220–1380) of the Vatnahverfi region between the late-fourteenth and early-fifteenth centuries (Ledger *et al.* 2014a, 2014b). The abandonment of farms across the Vatnahverfi region support Christian Madsen’s (2014) hypothesis that settlement became increasingly concentrated on farms on the inner and middle fjords, as distant and less productive upland and coastal regions were gradually abandoned (Fig. 5).

This refocus of settlements across the inner and middle fjords may have been an attempt to reduce distance between farms sharing labour for communal hunting and to re-settle in areas with more productive infields (Vésteinsson *et al.* 2002; Madsen 2014). Mid-fjord church sites at Hvalsey and Narsarsuaq appear to thrive from the fourteenth century at a time when farms in the Vatnahverfi area were abandoned (Madsen 2014), suggesting that resettlement concentration strategies were serving their purpose. But while this would have reduced inter-farm isolation it would also have entailed sustaining investment in fixed infrastructure on the inner fjord, which would have enhanced vulnerability to wild food shortages by increasing the distance to and from migratory seal populations beyond the outer fjord (Ogilvie *et al.* 2009). A shorter active season, less familiar conditions for hunting, and increased sea-ice—making boat transportation difficult and dangerous—would have compounded risks to food shortage.

### Mixed Resource Use Reduced Energetic Returns

NCT takes us beyond simplified theories of human behaviour and human-environment interaction to explain the role culture plays in modifying selective environments and the organisms within it (O’Brien and Laland 2012). Humans are seen to have the agency to consciously enhance their environments by selecting and modifying plants and animals to increase their biological and physical fitness (Zeder 2016). As discussed earlier, culture holds an important role accumulating and transmitting this information for future generations in a complex adaptive repertoire (Boyd *et al.* 2011). Culture operates at the group scale as a system of shared values, beliefs, and symbolic practices. As explained above, for the in Norse and Thule Inuit (Table 1), their systems of beliefs and practices were central to the regulation of their respective ‘cultural niches.’ Culture, and more specifically identity, had a significant role structuring knowledge of the local environment, its resources, and how to make use of them. The Norse identity has been elucidated, in part, from the material assemblages recovered from farms and church burials in Greenland’s Eastern and Western Settlements. Game pieces, toy boats, religious iconography, wooden tools, woollen clothing and metal jewellery reveal economic networks with the Scandinavian homelands and the North Atlantic islands and explain how the Norse viewed themselves as European farmers (Arneborg 2000).

The environment of southwest Greenland was, for the Norse, identified for modification into cultural landscapes. Reference to land is common to Norse written culture as a symbol of status and power (Jesch 2015). *The Kings Mirror* and *Erik the Red’s Saga* both draw attention to rich pastureland in Greenland’s settlement areas. Subsistence would have been organised according to seasonal activities, such as the growing/grazing season, harvest, and overwinter livestock stalling (McGovern 1980). This structured seasonal round was iteratively adjusted to different ecologies across the North Atlantic (Dugmore *et al.* 2005, 2007a). Levi-Strauss (1966) referred to cultural and symbolic structures as ‘totemic operators’—translating cognitive cultural structures into structured interactions with the environment. Cultural structures can be understood as practical ‘schemata’ that filter experience and legitimise action (Dugmore *et al.* 2012). Taking this point further, Adger *et al.* (2009) recognise that cultural identity provides not only legitimacy to act, but also directs the goals of adaptation. The socialised agricultural niche can be understood as an important component sustaining the Norse identity in Greenland (cf. Adger *et al.* 2013). Adaptive strategies would have been planned with the goal of sustaining agricultural production, which in turn limited their capacity to enhance energetic returns from hunting and foraging.

**Fig. 6:**
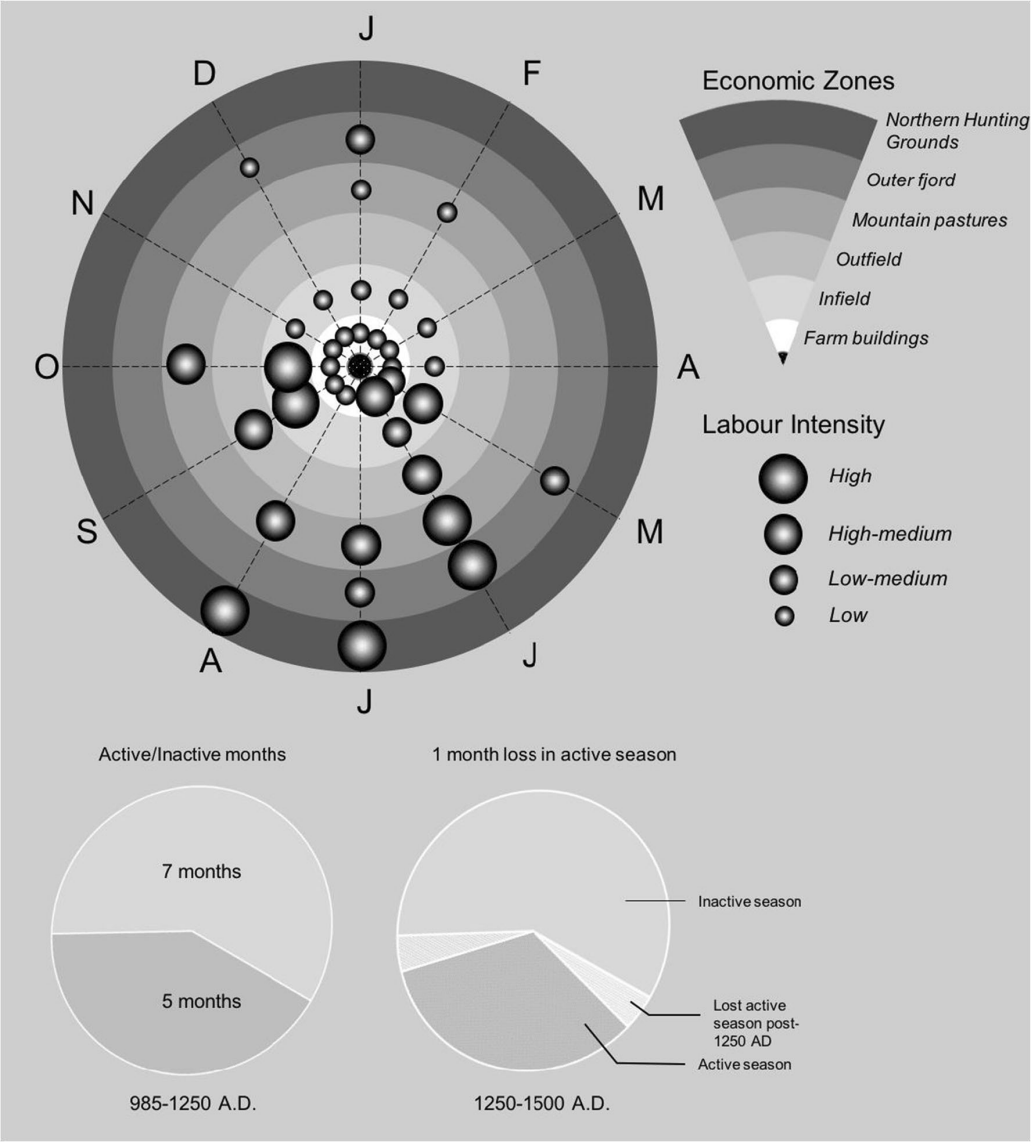
A theoretical model of distributed labour intensity across space over months of the year. Combining McGovern’s (1980) *seasonal round* of subsistence tasks with Øye’s (2013) standardised model of Norse farming, this model distinguishes the spatial intensity of labour between the seasons. This demonstrates the associative intensity of activities between the relatively inactive winter months and the laborious summer months. The months from November to April would have been largely inactive months other than small-scale seal hunting on the outer-fjord and attendance to overwintering livestock in byres and the infield. May to October were highly active involving process of transhumance whereby livestock were transported from byre and infield to outfield and mountain pastures as the summer progressed. Caribou hunting would also have taken place on across mountain pastures between settlements on the inner-fjord. Seal hunting took place primarily between the months of May and July. From July to late-August hunters would have taken to the northern hunting grounds in the Disko Bay region. This intense scheduling throughout the summer months is likely to have increasingly stressed the small Norse population throughout settlement. As the pie charts (below) show loss of a single month, possibly as a result of extended winters would have drastically disrupted the seasonal round and intensified task orientation

Simplified explanatory frameworks for human behaviour, such as Optimum Foraging Theory (OFT; cf. Codding and Bird 2015), are thus insufficient to explain the goal-oriented behaviour of the Norse farmers. In temperate climates, such as Scandinavia, the majority of activities operated within the farming core, but in Greenland, dietary records indicate a shifting dependence from core to peripheral locations, such as the outer fjord and uplands (Fig. 6). Intense scheduling in the summer months would have stressed the capacity of a small population to hunt resources efficiently from farms on the inner reaches of the fjord. As discussed, the settlement became increasingly concentrated on the inner fjord (Fig. 5). Increasing the distance between settlements and resource spaces would have caused energetic returns from hunting to decline, as hunters were required to travel greater distances to locate, catch, refine and return with seals from outer-fjord environments. (Zeder 2016: 329) summarised the potential trade-offs of investing in cultural niche construction:

“Investment in the management of a [domestic] species depends on the returns and demands of other potential resources, technological capacities, distribution of humans and resources across the landscape, as well as the physiological capacity to utilise these resources.”

In the last 40 years, archaeological studies across the North Atlantic islands have uncovered significant evidence to suggest the Norse were flexible to the impacts of climate change on their subsistence. This was also the case in Greenland. However, here strategies remained oriented towards supporting an unstable farming niche.

### Conjuncture: Synergisms between Economy and Climate

Mixed agriculture and hunting made the Norse subsistence system flexible to interannual variations in fodder production, but also limited their capacity to adopt more specialised strategies (Dugmore *et al.* 2012). Because the Norse were able to make incremental changes to their ‘cultural niche’ without compromising their identity, they can be said to have exhibited resilience. However, as Halstead and O’Shea (1989: 1) stipulated, “Culture endows man with exceptional flexibility in coping with his surroundings … [but] should not mask the fact that an effective strategy must match, in both capacity and scale, the variability with which it is to cope.” As the PAGES 2k climate model has shown, from the late-thirteenth century the North Atlantic entered a protracted cold period (PAGES 2k Consortium 2013). This had the effect of reducing homefield production and increasing livestock mortality on Norse farms (McGovern *et al.* 2014). Dietary proxies show the Norse adjusting to declining homefield yields by substituting farming shortfalls with increased marine mammal hunting (Arneborg *et al.* 2012; Smiarowski *et al.* 2017).

GISP2 data indicate the onset of more volatile and unpredictable climatic conditions from the early-fifteenth century (Dugmore *et al.* 2007b). The onset of climatic conditions that deviated from average cycles of variations made predictions about timing of farm activities and spring seal hunting increasingly difficult. The onset of acute climate variability also coincided with Greenland’s isolation from European markets (Dugmore *et al.* 2012), which underwent rapid transformation in the thirteenth and fourteenth centuries. The marginalisation of Norwegian political-economic power and the concentration of the stockfish and cloth trade in English and Northern German Hansa port towns resulted in the stagnation and decline of the North Atlantic ivory trade (Frei *et al.* 2015; Barrett 2018). The absence of alternative commodities for export to Europe isolated the Norse Greenlanders from trading partners, and the iron, clothing, and status imports they had long supplied (Dugmore *et al.* 2013). The synergisms between the impacts of climate uncertainty on subsistence and economic isolation from Europe would have undermined attempts to sustain agricultural economies as management of home-field production, livestock herding, and resources vital to technologies for tool making and manufacture of clothing became all but impossible.

## Conclusions

The Norse adapted to the environments of southwest Greenland through a combination of landscape learning and culturally transmitted knowledge. During the settlement period information about resource location, timing, and sustainable limits would have been accumulated to refine livestock assemblages and organise seal and caribou hunting (Rockman 2009). The Norse proved flexible to changing climates—refining livestock ratios and increasing the proportion of their diet from non-migratory seals (Arneborg *et al.* 2012; Fig. 3). This strategy was sustained until the mid-fifteenth century, when settlement came to an end.

Agricultural instability and climate uncertainty is a likely cause of social-ecological disequilibrium. The Norse made incremental changes to their diet, tools, buildings, clothing, and field systems without discernible impacts on their identity as Scandinavian-European farmers. These changes were sufficient to sustain subsistence in the mild conditions of the MCA (*c.* AD 950–1250). The onset of cooler climatic conditions from the late-thirteenth century would have increased stress on homefield production, and subsequently stretched the application of hunting strategies. The construction of a farming niche would have distinguished between a socialised inside and a hostile outside (Arneborg 1997). Culturally transmitted information, accumulated in temperate Norway and other North Atlantic islands, was ill-equipped to respond to unpredictable interannual climate variation. Economic behaviours were dependent on suitable conditions for home-field production and animal rearing. Norse behaviours—associated with cultural and ecological reliance on sedentary animal husbandry—thus reached their adaptive limits in the late-Medieval climates of Southwest Greenland. The Norse agricultural systems were thus mismatched with the scale of climate variation.

## References

[CR1] Adderley PW, Simpson IA (2006). Soils and palaeo-climate based evidence for irrigation requirements in Norse Greenland. Journal of Archaeological Science.

[CR2] Adger NW, Dessai S, Goulden M, Hulme M, Lorenzoni I, Nelson DR, Naess LO, Wolf J, Wreford A (2009). Are there social limits to adaptation to climate change?. Climatic Change.

[CR3] Adger N, Barnett J, Brown K, Marshall N, O’Brien K (2013). Cultural dimensions of climate change impacts and adaptation. Nature Climate Change.

[CR4] Appelt M, Gulløv HC, Maschner H, Mason O, McGhee R (2009). Tunit, Norsemen, and Inuit in thirteenth-century Northwest Greenland—Dorset between the devil and the Deep Sea. The northern world, AD 900–1400.

[CR5] Armstrong CG, Shoemaker AC, McKechnie I, Ekblom A, Szabó P, Lane PJ, McAlvay AC, Boles OJ, Walshaw S, Petek N, Gibbons KS, Morales EQ, Anderson EN, Ibragimow A, Podruczny G, Vamosi JC, Marks-Block T, LeCompte JK, Awâsis S, Nabess C, Sinclair P, Crumley CL (2017). Anthropological contributions to historical ecology: 50 questions, infinite prospects. PLoS ONE.

[CR6] Arneborg J, Gilberg R, Gulløv HC (1997). Cultural Borders: Reflections on Norse-Eskimo interaction. *Fifty Years of Arctic Research. Anthropological Studies from Greenland to Siberia*.

[CR7] Arneborg J, Fitzhugh WW, Ward EI (2000). Greenland and Europe. *Vikings: The North Atlantic Saga*.

[CR8] Arneborg J, Barrett J (2003). Norse Greenland: Reflections on settlement and depopulation. Contact, continuity, and collapse: The Norse colonization of the North Atlantic.

[CR9] Arneborg J (2005). Greenland irrigation systems on a west Nordic background. Water Management in Medieval Rural Economy. Ruralia.

[CR10] Arneborg J, Brink S, Price N (2008). The Norse Settlements in Greenland. The Viking World.

[CR11] Arneborg J, Lynnerup N, Heinmeier J, Møhl J, Rud N, Sveinbjörnsdóttir ÁE (2012). Norse Greenland dietary economy ca. 980-ca. AD 1450: Introduction. Journal of the North Atlantic.

[CR12] Barnett JH, Barnett JH, Gibbon SJ (2015). Maritime Societies and the Transformation of the Viking Age and Medieval World. Maratime Societies of the Viking and Medieval World.

[CR13] Barrett JH, Gerrard C, Gutiérrez A (2018). Medieval fishing and fish trade. *The Oxford Handbook of Later Medieval Archaeology in Britain*.

[CR14] Bentley AR, O’Brien MJ (2015). Collective behavior, uncertainty and environmental change. Philosophical Transactions of the Royal Society A.

[CR15] Berkes F (2017). Sacred ecology.

[CR16] Bill J, Brink S, Price N (2008). Ships and the Sea. The Viking World.

[CR17] Blonder B, Moulton DE, Blois J, Enquist BJ, Graae BJ, Macias-Fauria M, McGill B, Nogué S, Ordonez A, Sandel B, Svenning J-C (2017). Predictability in community dynamics. Ecology Letters.

[CR18] Boyd R, Richerson PJ, Henrich J (2011). The cultural niche: Why social learning is essential for human adaptation. Proceedings of the National Academy of Sciences.

[CR19] Brace C, Geoghegan H (2011). Human geographies of climate change: Landscape, temporality and lay knowledge. Progress in Human Geography.

[CR20] Brody H (2001). The other side of Eden.

[CR21] Buckland P, Brink S, Price N (2008). The North Atlantic farm: an environmental view. The Viking World.

[CR22] Buckland PC, Edwards KJ, Panagiotakopulu E, Schofield JE (2009). Palaeoecological and historical evidence for manuring and irrigation at Gardar (Igaliku), Norse eastern settlement, Greenland. Holocene.

[CR23] Butzer KW (2012). Collapse, environment and society. PNAS.

[CR24] Church MJ, Dugmore AJ, Mairs K-A, Millard AR, Cook GT, Sveinbjarnardottir G, Ascough PA, Roucoux KH (2007). Charcoal production during the Norse and Early Medieval periods in Eyjafjallahreppur. southern Iceland. Radiocarbon.

[CR25] Codding B-F, Bird DW (2015). Behavioural ecology and the future of archaeological science. Journal of Archaeological Science.

[CR26] Crumley C (1994). Historical ecology.

[CR27] Cumming GS, Barnes G, Southworth J, Norberg J, Cumming GS (2008). Environmental Asymmetries. Complexity theory for a sustainable future.

[CR28] Diamond J (2005). Collapse: How societies choose to fail or survive.

[CR29] Dugmore AJ, Church MJ, Buckland PC, Edwards KJ, Lawson I, McGovern TH, Panagiotakopulu E, Simpson IA, Skidmore P, Sveinbjarnardóttir G (2005). The Norse *landnám* on the North Atlantic islands: An environmental impact assessment. Polar Record.

[CR30] Dugmore, A. J., Church, M. J., Mairs, K.-A., McGovern, T. H., Newton, A. J., and Sveinbjarnardottir, G. (2006). An over-optimistic pioneer fringe? Environmental perspectives on medieval settlement abandonment in Orsmork, Southern Iceland. In Dynamics of nothern societies: proceedings of the SILA/NABO conference on Arctic and North Atlantic Archaeology, Copenhagen, May 10th-14th, 2004. CopenhagenL, Aarhus University Press, pp. 335–345.

[CR31] Dugmore AJ, Keller C, McGovern TH (2007). The Norse Greenland settlement: Reflections on climate change, trade and the contrasting fates of human settlements in the Atlantic islands. Arctic Anthropology.

[CR32] Dugmore AJ, Borthwick DM, Dawson A, Edwards KJ, Keller C, Mayewski P, McGovern TH, Mairs K, Sveinbjarnardóttir G (2007). The role of climate in settlement and landscape change in the North Atlantic Islands: An assessment of cumulative deviations in high-resolution proxy climate records. Human Ecology.

[CR33] Dugmore AJ, Keller C, McGovern TH, Casely A, Smiarowski K, Adger NW, Lorenzoni I, O’Brien KL (2009). Norse Greenland settlement and limits to adaptation. Adapting to climate change.

[CR34] Dugmore AJ, Casely A, Keller C, McGovern TH, Anderson A, Barrett J, Boyle K (2010). Conceptual Model of Seafaring, Climate and Early European Exploration and Settlement of the North Atlantic Islands. Global Origins and Development of Seafaring.

[CR35] Dugmore AJ, McGovern TH, Vésteinsson O, Arneborg J, Streeter R, Keller C (2012). Cultural adaptation, compounding vulnerabilities and conjunctures in Norse Greenland. Proceedings of the National Academy of Sciences.

[CR36] Dugmore AJ, McGovern TH, Streeter R, Madsen CK, Smiarowski K, Keller C, Sygna L (2013). ‘Clumsy Solutions’ and ‘Elegant Failures’: Lessons on Climate Change Adaptation from the Settlement of the North Atlantic. A changing environment for human security: Transformative approaches to research, policy and action.

[CR37] Enghoff IB (2003). Hunting, fishing and animal husbandry at the farm beneath the sand, western settlement. Meddelelser om Grønland: Man and Society.

[CR38] Epstein SA (2009). An economic and social history of later medieval Europe, 1000–1500.

[CR39] Frei KM, Coutu AN, Smiarowski K, Harrison R, Madsen CK, Arneborg J, Frei R, Gudmundsson G, Sindbæk SM, Woollett J, Hartman S, Hicks M, McGovern TH (2015). Was it for walrus? Viking age settlement and medieval walrus ivory trade in Iceland and Greenland. World Archaeology.

[CR40] Gardela L (2012). What the Vikings did for fun? Sports and pastimes in medieval northern Europe. World Archaeology.

[CR41] Golding KA, Simpson IA, Schofield JE, Edwards KJ (2011). Norse-Inuit interaction and landscape change in southern Greenland. A geochronological, pedological and palynological investigation. Geoarchaeology.

[CR42] Golding KA, Simpson IA, Wilson CA, Low EC, Schofield JE (2015). Europeanization of sub-Arctic environments: Perspectives from Norse Greenland’s outer fjords. Human Ecology.

[CR43] Gullov HC (2004). Gronlands Forhistorie.

[CR44] Gulløv HC (2008). The nature of contact between native Greenlanders and Norse. Journal of the North Atlantic.

[CR45] Halstead P, O'Shea J (1989). Bad year economics: Cultural responses to risk and uncertainty.

[CR46] Hayeur-Smith M (2013). Thorir's bargain: gender, vadmal and the law. World Archaeology.

[CR47] Hayeur-Smith M (2014). Dress, cloth, and the Farmer’s wife: Textiles from Ø172 Tatsipataa, Greenland, with comparative data from Iceland. Journal of the North Atlantic.

[CR48] Hayeur-Smith M, Arneborg J, Smith KP (2016). The 'Burgundian' hat from Herjolfsnes, Greenland: new discoveries, new dates. Danish Journal of. Archaeology.

[CR49] Høegsberg, M. (2014) ‘Islands across the sea – Aspects of Regionality in the Norse North Atlantic diaspora.’ In Kristiansen, M.S. and Giles, K. (Eds.) Dwelling, identities and homes: European housing culture from the Viking age to the renaissance. Jutland Archaeological Society.

[CR50] Howard JB, Scarborough VL, Isaac BL (1993). A Paleohydraulic approach to examining agricultural intensification in Hohokam irrigation systems. Research in economic anthropology.

[CR51] Ingold T (2000). The perception of the environment: Essays in livelihood, dwelling and skill.

[CR52] Ingram S (2008). Streamflow and population change in the lower Salt River valley of Arizona, ca. A.D. 775 to 1450. American Antiquity.

[CR53] Jensen KG, Kuijpers A, Koç N, Heinemeier J (2004). Diatom evidence of hydrographic changes and ice conditions in Igaliku Fjord, South Greenland, during the past 1500 years. The Holocene.

[CR54] Jesch J (2015). The Viking diaspora.

[CR55] Keller, C. (1989). The Eastern Settlement Reconsidered. Some analysis of Norse Medieval Greenland. University of Oslo, Unpublished PhD Thesis.

[CR56] Kendal D (2011). Cultural niche construction and human learning environments: Investigating socio-cultural perspectives. Biological Theory.

[CR57] Kennett DJ, Marwan N (2015). Climate volatility, agricultural uncertainty, and the formation, consolidation and breakdown of preindustrial agrarian states. Philosophical Transactions of the Royal Society A.

[CR58] Kopar L (2009). The Use of Artistic Media in Norse Greenland. Journal of the North Atlantic.

[CR59] Kuijpers A, Mikkelsen N, Ribeiro S, Seidenkrantz M-S (2014). Impact of medieval fjord hydrography and climate on the western and eastern settlements in Norse Greenland. Journal of the North Atlantic.

[CR60] Laland KN, Brown GR (2006). Niche construction, human behavior, and the adaptive-lag hypothesis. Evolutionary Anthropology.

[CR61] Laland KN, Brown GR (2011). Sense and Nonsense.

[CR62] Laland KN, Odling-Smee FJ, Feldman MW (1999). Evolutionary consequences of niche construction and their implications for ecology. PNAS.

[CR63] Lavigne F, Degeai J-P, Komorowsku JC, Guillet S, Robert V, Lahitte P, Oppenheimer C, Stoffel M, Vidal CM, Surono S, Pratomo I, Wassmer P, Hajdas I, Hadmoko DS, De Belizal E (2013). Source of the great AD 1257 mystery eruption unveiled Samalas volcano, Rinjani volcanic complex, Indonesia. PNAS.

[CR64] Ledger PM, Edwards KJ, Schofield JE (2013). Shieling activitiy in the Norse eastern settlement: Palaeoenvironment of the ‘mountain farm’, Vatnahverfi, Greenland. The Holocene.

[CR65] Ledger PM, Edwards KJ, Schofield JE (2014). Vatnahverfi: A green and pleasant land? Palaeoecological Reconstructions of Environmental and Land-use Change. Journal of the North Atlantic.

[CR66] Ledger PM, Edwards KJ, Schofield JE (2014). A multiple profile approach to the palynological reconstruction of Norse landscapes in Greenland’s eastern settlement. Quaternary Research.

[CR67] Levi-Strauss C (1966). The Savage Mind.

[CR68] Lynnerup N (2014). Endperiod demographics of the Greenland Norse. Journal of the North Atlantic.

[CR69] Madsen, C.K. (2014) *Pastoral Settlement, Farming, and Hierarchy in Norse Vatnahverfi, South Greenland.* PhD Thesis. University of Copenhagen.

[CR70] Mason OT (2007). North American bows and quivers.

[CR71] McGovern TH (1980). Cows, harp seals, and churchbells: Adaptation and extinction in Norse Greenland. Human Ecology.

[CR72] McGovern TH, Green S, Perlman S (1985). The Arctic frontier of Norse Greenland. *The Archaeology of Frontiers and Boundaries*.

[CR73] McGovern, T. H. (1991). Climate, correlation, and causation in Norse Greenland. Arctic Anthropology: 77–100.

[CR74] McGovern TH, Crumley CL (1994). Management for extinction in Norse Greenland. *Historical Ecology: Cultural Knowledge and Changing Landscapes*.

[CR75] McGovern TH, Bigelow G, Amorosi T, Russell D (1988). Northern Islands, human error, and environmental degradation: A preliminary model for social and ecological change in the medieval North Atlantic. Human Ecology.

[CR76] McGovern TH, Fitzhugh WW, Ward EI (2000). The Demise of Norse Greenland. Vikings: The North Atlantic Saga.

[CR77] McGovern TH, Harrison R, Smiarowski K, Harrison R, Maher RA (2014). Sorting sheep & goats in medieval Iceland and Greenland: Local subsistence or world system. Long-term human Ecodynamics in the North Atlantic: An archaeological study.

[CR78] McKay, N.P. and Kaufman, D.S. (2014) ‘An extended Arctic proxy temperature database for the past 2,000 years’. Nature Scientific Data 1:140026 10.1038/sdata.2014.26. 10.1038/sdata.2014.26 PMC4322576 25977783

[CR79] Meaks N, Cartwright CR, King JC, Pauksztat B, Storrie R (2005). Caribou and seal hair: Examining by scanning electron microscope. Arctic clothing.

[CR80] Mehl IK, Hjelle KL (2017). From pollen percentage to regional vegetation cover - A new insight into cultural landscape development in western Norway. Review of Palaeobotany and Palynology.

[CR81] Middleton GD (2017). Understanding collapse: Ancient history and modern myths.

[CR82] Miller GH, Geirsdóttir Á, Zhong Y, Larsen D, Otto-Bliesner B, Holland MM, Bailey DA, Refsnider KA, Lehman SJ, Southon JR, Anderson C, Björnsson H, Thordarson T (2012). Abrupt onset of the little ice age triggered by volcanism and sustained by sea-ice/ocean feedbacks. Geophysical Research Letters.

[CR83] Moltke I, Fumagalli M, Korneliussen TS, Crawford JE, Bjerregaard P, Jørgensen ME, Grarup N, Gulløv HC, Pedersen O, Hansen T, Neilsen R, Albrechtsen A (2015). Uncovering the genetic history of the present-day Greenlandic population. American Journal of Human Genetics.

[CR84] Myhre B, Almås R (2004). Agriculture, landscape and society, ca. 4000 BC-AD 800. Norwegian agricultural history.

[CR85] Nedkvitne A (2014). The German Hansa and Bergen 1100-1600.

[CR86] Nelson MC, Hegmon M, Kintigh KW, Kinzig AP, Nelson BA, Anderies JM, Abbott DA, Spielmann KA, Ingram SE, Peeples MA, Kulow S, Strawhacker CA, Meegan CA, Cooper J, Sheets P (2012). Long-term Vulnerability and Resilience: Three Examples from Archaeological Study in the Southwestern United States and Northern Mexico. Surviving Sudden Environmental Change: Answers from Archaeology.

[CR87] Nelson MC, Ingram SE, Dugmore AJ, Streeter R, Peeples MA, McGovern TH, Hegmon M, Spielmann KA, Simpson IA, Strawhacker C, Comeau LE, Torvinen A, Madsen CK, Hambrecht G, Smiarowski K (2016). Climate changes, vulnerabilities, and food security. PNAS.

[CR88] Normand S., Randin C., Ohlemuller R., Bay C., Hoye T. T., Kjaer E. D., Korner C., Lischke H., Maiorano L., Paulsen J., Pearman P. B., Psomas A., Treier U. A., Zimmermann N. E., Svenning J.-C. (2013). A greener Greenland? Climatic potential and long-term constraints on future expansions of trees and shrubs. Philosophical Transactions of the Royal Society B: Biological Sciences.

[CR89] O'Brien MJ, Laland KN (2012). Genes, Culture, and Agriculture: an Example of Human Niche Construction. Current Anthropology.

[CR90] Odling-Smee J, Erwin DH, Palkovacs EP, Feldman MW, Laland KN (2013). Niche constriction theory: A practical guide for ecologists. The Quarterly Review of Biology.

[CR91] Ogilvie AEJ, Woollett JM, Smiarowski K, Arneborg J, Troelstra S, Kuijpers A, Pálsdóttir A, McGovern TH (2009). Seals and sea ice in medieval Greenland. Journal of the North Atlantic.

[CR92] Østergård E (2004). Woven into the earth: Textiles from Norse Greenland.

[CR93] Østergård E (2011). Medieval Garments Reconstructed: Norse Clothing Patterns.

[CR94] Øye I, Almås R (2004). Agricultural conditions and rural societies ca. 800-1350 – An introduction. *Norwegian Agricultural History*.

[CR95] Øye I (2009). Settlement patterns and field Systems in Medieval Norway. Landscape History.

[CR96] Øye I, Kerig T, Zimmermann A (2013). Technology, land use and transformation in Scandinavian landscapes, *c.* 800-1300 AD. Economic archaeology: From structure to performance in European archaeology.

[CR97] PAGES 2k Consortium (2013). Continental-scale temperature variability during the last two millennia. Nature Geoscience.

[CR98] Park RW (2005). Growing up north: Exploring the archaeology of childhood in Thule and Dorset cultures of Arctic Canada. Archaeological Papers of the American Athropological Association.

[CR99] Perdikaris S (1999). From chiefly provisioning to commercial fishery: Long-term economic exchange in Arctic Norway. World Archaeology.

[CR100] Perdikaris S, McGovern TH, Rick TR, Erlandson JM (2008). Codfish and kings, seals and subsistence: Norse marine resource use in the North Atlantic. Viking Voyagers.

[CR101] Raghavan M, DeGiorgio M, Albrechtsen A, Moltke I, Skoglund P, Korneliussen TS, Grønnow B, Appelt M, Gulløv HC, Friesen TM, Fizhugh W, Malström H, Rasmussen S, Olsen J, Melchior L, Fuller BJ, Farhni SM, Stafford T, Grimes V, Renouf MAP, Cybulski J, Lynnerup N, Lahr MM, Britton K, Knecht R, Arneborg J, Metspalu M, Cornejo OE, Malaspinas A-S, Wang Y, Rasmussen M, Raghvana V, Hansen TVO, Khusnutdinova E, Pierre T, Dneprovsky K, Andreasen C, Lange H, Hayes MG, Coltrain J, Spitsyn VA, Götherström A, Orlando L, Kivisild T, Villems R, Crawford MH, Nielsen FC, Dissing J, Heinemeier J, Meldgaard M, Bustamante C, O’Rourke DH, Jakobsson M, Gilbert MT, Nielsen R, Willerslev E (2014). The genetic prehistory of the New World Arctic. Science.

[CR102] Reinton, L. (1960). Til seters, Samlaget, Oslo.

[CR103] Riede F, McCartan S (2009). Climate change, demography and social relations: An alternative view of the late Palaeolithic pioneer colonization of southern Scandinavia. Mesolithic horizons: Papers presented at the seventh international conference on the Mesolithic in Europe, Belfast.

[CR104] Riede F (2011). Adaptation and niche construction in human prehistory: A case study from the southern Scandinavian late glacial. Philosophical Transactions of the Royal Society B.

[CR105] Riede F, Berge R, Jasinski ME, Sognnes K (2012). Theory for the A-theoretical: Niche construction theory and its implications for environmental archaeology. N-TAG TEN. Proceedings of the 10th Nordic TAG conference at Stiklestad, Norway 2009.

[CR106] Riede, F., and Pedersen, J. B. (2018). Late Glacial human dispersal in northern Europe and disequilibrium dynamics. Human Ecology. 10.1007/s10745-017-9964-8.

[CR107] Riede F, Johannsen NN, Högberg A, Nowell A, Lombard M (2018). The role of play objects and object play in human cognitive evolution and innovation. Evolutionary Anthropology.

[CR108] Rockman M, Rockman M, Steele J (2003). Knowledge and learning in the archaeology of colonization. Colonization of unfamiliar landscapes: The archaeology of adaptation.

[CR109] Rockman M (2009). A world with a new sky: Climate variability, environmental expectations, and the historical colonization of eastern North America. Historical Archaeology.

[CR110] Roesdahl E (2016). The Vikings.

[CR111] Rowley-Conwy P, Colledge S, Conolly J, Dobney K, Manning K, Shennan S (2013). North of the frontier: Early domestic animals in northern Europe. The Origins and Spread of Domestic Animals in Southwest Asia and Europe.

[CR112] Rowley-Conwy P, Layton R (2011). Foraging and farming as niche construction: Stable and unstable adaptations. Philosophical Transactions of the Royal Society B.

[CR113] Roussell A (1941). Farms and Churches in the Medieval Norse Settlements of Greenland. Meddelelser om Grønland.

[CR114] Sandvik G, Sigurdsson JV, McTurk R (2005). Laws. A companion to old Norse-Icelandic literature and culture.

[CR115] Seaver KA (1996). The Frozen Echo: Greenland and the Exploration of North America, ca. A.D. 1000-1500.

[CR116] Scott GR, Jolie RB (2008). Tooth-tool use and yarn production in Norse Greenland. Alaska Journal of Anthropology.

[CR117] Scott GR, Halffman CM, Pedersen PO (1992). Dental conditions of medieval Norsemen in the North Atlantic. Acta Archaeologica.

[CR118] Smiarowski K, Harrison R, Brewington S, Hicks M, Hérbert FJ, Prehal B, Hambrecht G, Woollett J, McGovern TH, Albarella U (2017). Zooarchaeology of the Scandinavian settlements in Iceland and Greenland: Divergent pathways. Oxford handbook of Zooarchaeology.

[CR119] Smith KP (1995). Landnam: The Settlement of Iceland in Archaeological and Historical Perspective. World Archaeology.

[CR120] Smith BD (2011). General patterns of niche construction and the management of ‘wild’ plant and animal resources by small-scale pre-industrial societies. Philosophical Transactions of the Royal Society B.

[CR121] Sveinbjarnardottir G (1991). Shielings in Iceland, an archaeological and historical survey. Acta Archaeologica.

[CR122] Svenning J-C, Sandel B (2013). Disequilibria vegetation dynamics of widespread forest plant species in nemoral Europe. Ecography.

[CR123] Svenning JC, Eiserhardt WL, Normand S, Ordonez A, Sandel B (2015). The Influence of Palaeoclimate on Present-Day Patterns in Biodiversity and Ecosystems. Annual Review of Ecology, Evolution and Systematics.

[CR124] Tainter JA (1988). The Collapse of Complex Societies.

[CR125] Terrel JE, Hart JP, Barut S, Cellinese N, Curet A, Denham T, Kusimba CM, Latinis K, Oka R, Palka J, Pohl MED, Pope KO, Williams PR, Haines H, Staller JE (2003). Domesticated landscapes: The subsistence ecology of plants and animal domestication. Archaeological Method and Theory.

[CR126] Thompson M, Sygna L, O’Brien K, Wolf J (2013). Clumsy solutions to environmental change: Lessons from cultural theory. A changing environment for human security: Transformative approaches to research, policy and action.

[CR127] Vésteinsson O (2009). Parishes and communities in Norse Greenland. Journal of the North Atlantic.

[CR128] Vésteinsson O, Sverrisdottir B (2006). The Building and its Context. Reykjavik 871 ± 2: The Settlement Exhibition.

[CR129] Vésteinsson O, McGovern TH, Keller C (2002). Enduring impacts: Social and environmental aspects of Viking age settlement in Iceland and Greenland. Archaeologia Islandica.

[CR130] Zeder M (2009). The Neolithic macro-(R)evolution: Macroevolutionary theory and the study of culture change. Journal of Archaeological Research.

[CR131] Zeder M (2015). Core questions in domestication research. PNAS.

[CR132] Zeder M (2015). Reply to Mohlenhoff *et al*.: Human behavioral ecology needs a rethink that niche construction theory can provide. PNAS.

[CR133] Zeder M (2016). Domestication as a model system for niche construction theory. Evolutionary Ecology.

